# Pulmonary Arterial Hypertension: Pathophysiology and Treatment

**DOI:** 10.3390/diseases6020038

**Published:** 2018-05-16

**Authors:** Norris S. H. Lan, Benjamin D. Massam, Sandeep S. Kulkarni, Chim C. Lang

**Affiliations:** 1 School of Medicine and Pharmacology, University of Western Australia, Perth 6009, Australia; 21151851@student.uwa.edu.au (N.S.H.L.); 21139507@student.uwa.edu.au (B.D.M.); 21113058@student.uwa.edu.au (S.S.K.); 2 Division of Molecular and Clinical Medicine, Mailbox 2, Ninewells Hospital and Medical School, University of Dundee, Dundee DD1 9SY, UK

**Keywords:** pulmonary arterial hypertension, nitric oxide, prostacyclin-thromboxane, endothelin-1, phosphodiesterase-5 inhibitor, soluble guanylate cyclase stimulators, prostacyclin analogues, prostacyclin receptor agonists, endothelin receptor antagonists, mortality

## Abstract

Pulmonary arterial hypertension (PAH), the first category of pulmonary hypertension, is a chronic and progressive disorder characterised by angioproliferative vasculopathy in the pulmonary arterioles, leading to endothelial and smooth muscle proliferation and dysfunction, inflammation and thrombosis. These changes increase pulmonary vascular resistance and subsequent pulmonary arterial pressure, causing right ventricular failure which leads to eventual death if untreated. The management of PAH has advanced rapidly in recent years due to improved understanding of the condition’s pathophysiology, specifically the nitric oxide, prostacyclin-thromboxane and endothelin-1 pathways. Five classes of drugs targeting these pathways are now available: phosphodiesterase-5 inhibitors, soluble guanylate cyclase stimulators, prostacyclin analogues, prostacyclin receptor agonists and endothelin receptor antagonists. These developments have led to substantial improvements in mortality rate in recent decades. Recently, long-term studies have demonstrated sustained progression-free survival and have created a new paradigm of initial combination therapy. Despite these targeted therapies, PAH is still associated with significant morbidity and mortality. As such, further research into broadening our understanding of PAH pathophysiology is underway with potential of increasing the repertoire of drugs available.

## 1. Introduction

Pulmonary hypertension (PH) consists of a group of diseases with a resting mean pulmonary artery pressure (mPAP) ≥ 25 mmHg as measured with right heart catheterization [1]. Since 1998, major revisions in the classification of PH have categorised the disease based on the anatomical site and aetiology. The latest revision, in 2013, is shown in Table 1.

PH is considered a rare disease, with a population-based study in the Netherlands suggesting a prevalence rate of 2.6% based on echocardiographic findings [2]. According to one Australian cohort study, PH secondary to left heart disease was the subtype with the greatest prevalence but also conferred the highest mortality, followed by PH secondary to respiratory diseases [3]. 

Group 1 of PH is pulmonary arterial hypertension (PAH), which is defined as mPAP ≥ 25 mmHg, pulmonary artery wedge pressure (PAWP) ≤ 15 mmHg and pulmonary vascular resistance (PVR) > 3 Wood units [1]. PAH is further subcategorised according to its aetiology, with idiopathic PAH (iPAH) comprising the majority of cases, followed by PAH associated with connective tissue diseases (CTD) and congenital heart disease (see Table 1) [4]. In PAH, the pre-capillary arterioles are affected by an angioproliferative vasculopathy that increases the pulmonary vascular resistance, thereby increasing the right ventricular afterload with the resulting right heart failure being the ultimate cause of mortality [5]. When adequately treated, PAH exhibits the best prognosis when compared to other PH categories according to the aforementioned Australian study [3]. 

According to registries in the United Kingdom, the reported incidence and prevalence rates of PAH are 1.1–2.4 and 6.6–15.0 cases per million per year, respectively [6]. Contemporary data from the 2014 UK National Audit on Pulmonary Hypertension found that the median age of diagnosis of PAH was 60 years in females and 58 years in males, with more than 25% of patients being over 70 years—thereby refuting previous conceptions of PAH being a disease of the young [7]. Data from the United States-based registry to evaluate early and long-term pulmonary arterial hypertension disease management (REVEAL registry) demonstrated certain sex preponderances for specific subtypes of PAH, such as a greater female distribution in iPAH [8]. Males demonstrated a lower survival rate at both two and five years post-enrolment; however, subanalysis showed similar survival rates between women and men <60 years at enrolment but poorer survival rates in men >60 compared to women [8]. Overall median survival rates have improved dramatically over the past two decades (from 2.8 to 7 years in the aforementioned American registry), presumably due to a combination of significant advances in treatment strategies and patient support strategies [9]. 

## 2. Risk and Prognostic Factors of PAH

Drugs, toxins, CTD and certain infections (such as HIV and schistosomiasis) have been strongly associated with PAH, and have thus appeared as subcategories in the classification [10,11]. The drugs and toxins considered as definite risk factors include aminorex, fenfluramine, dexfenfluramine and selective serotonin reuptake inhibitors (SSRIs) [10]. In particular, iPAH has been found to be strongly associated with female gender, family history and genetic variants, especially bone morphogenetic protein receptor type 2 (*BMPRII*) mutations [4,12]. 

Prognostic stratification of PAH in clinical practice requires extensive assessment and investigation at specialised PH centres. As highlighted in Table 2, the 2015 ESC/ERS (European Society of Cardiology and the European Respiratory Society) guidelines recommend a series of variables to stratify patients into low, intermediate and high-risk categories (corresponding to an estimated one-year mortality rate of <5%, 5–10% and >10% respectively) which subsequently guides management [13]. These variables include an assessment of clinical function, World Health Organisation functional class (WHO-FC), exercise capacity and right ventricular function, with the WHO-FC segregating PAH patients into four categories based on their physical capacity impairment and dyspnea [13,14]. These variables are measured through a combination of imaging (echocardiography, cardiac magnetic resonance imaging), haemodynamics (e.g., right atrial pressure, cardiac index, mixed venous oxygen saturation), exercise testing (e.g., 6-minute walking test, cardiopulmonary exercise testing) and biochemical markers (markers of vascular dysfunction, myocardial stress, low cardiac output and secondary organ damage) [13,14]. Other factors such as age, sex, PAH subtype and symptomatic features of heart failure should be considered in prognostic evaluation, although their importance is largely determined on a case-by-case basis supplemented with clinical expertise [13]. 

## 3. Genetics

Heritable PAH (hPAH), a subcategory of PAH, exhibits an autosomal dominant pattern of inheritance, with several associated germline gene mutations having been identified [15]. These mutations primarily occur in the genes encoding the transforming growth factor β (TGF-β) receptor superfamily, namely *BMPRII* [16], activin receptor-like kinase 1 (*ALK1*) [17], mothers against decapentaplegic homolog 9 (*SMAD9*) and endoglin 1 (*ENG*) [16,17,18]. Approximately 70% of hPAH patients [15], and a minority (10–40%) of patients with apparently sporadic iPAH [19], have mutations in the *BMPRII*; in comparison to mutations in *ALK 1*, *SMAD9* and *ENG*, which collectively appear in only 5% of the hPAP population [15,19,20]. Though hPAH was previously considered to exhibit genetic anticipation (i.e., earlier age of onset and death in consecutive generations), recent data has challenged this conclusion [21]. 

Diagnosis of hPAH is complicated by the incomplete penetrance of *BMPRII* mutations, with only 20% of individuals possessing disease-associated variants developing the condition [21]. Furthermore, the variable expressivity and female predominance of these gene variants reveal the combination of genetic, genomic and environmental factors in PAH pathogenesis [21,22].

The most commonly studied gene mutation in relation to PAH pathogenesis is with *BMPRII*. Animal models demonstrate that reduced *BMPRII* activity in pulmonary vascular endothelial cells increases the incidence of apoptosis, leading to vascular remodelling and ultimately PAH [23,24]. Additionally, improving *BMPRII* expression in mice models through microRNA inhibition limits endothelial dysfunction and attenuates hypoxia-induced PAH [25]. 

Though genetic testing for hPAH is available, this service should be offered by trained individuals to those patients with iPAH considered to be sporadic or induced by anorexigens and to patients with a family history of PAH [13]. Ethical principles of genetic testing must include, among others, preserving patient and family autonomy, avoiding harm, and allowing equal access to genetic counselling for all patients. As outlined previously, the variable penetrance and expressivity of the *BMPRII* mutations may cause genetic testing to identify variants of unknown clinical significance, thereby causing unnecessary anxiety. Nonetheless, genetic testing is available which involves initial testing of only *BMPRII* variants, with negative results prompting further investigation of rarer pathogenic mutations (e.g., *ALK1* and *ENG*) [13]. 

## 4. Pathophysiology

PAH may be idiopathic or secondary to various conditions, but regardless of the underlying aetiology, patients exhibit similar pathological changes which include enhanced pulmonary arteriole contractility, endothelial dysfunction, remodelling and proliferation of both endothelial and smooth muscle cells, and in situ thrombi [5]. The physiological outcome of these disturbances is the partial occlusion of small pulmonary arteries, eventuating in increased PVR, subsequent right ventricular failure and death [5]. 

**Figure 1:**
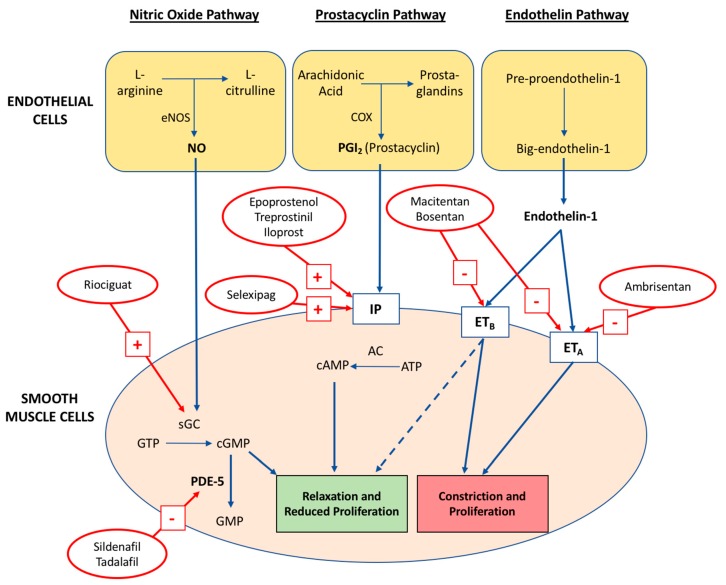
The key abnormal pathways targeted in the pharmacological treatment of pulmonary arterial hypertension and the mechanism of action for contemporary drugs. The dashed line from ET_B_ denotes action of endothelial ET_B_ activation via NO and PGI_2_ production. Adapted from Prior et al. MJA 2016 [28].

Underpinning these progressive pulmonary vascular defects is the disruption of three key signalling pathways outlined in Figure 1: nitric oxide (NO), prostacyclin (PGI_2_) and thromboxane A_2_ (TXA_2_), and endothelin-1 (ET-1) [26]. Broadly speaking, PAH is caused by impaired vasodilation from reduced PGI_2_ production (cyclooxygenase-2 dysregulation) and NO synthase (eNOS) function, with concurrent vasoconstrictive and mitogenic effects of an upregulated ET-1 signalling system [26,27]. A mechanistic understanding of these three pathways has prompted rapid development in the quantity and efficacy of targeted pharmacological therapies for PAH.

### 4.1. Nitric Oxide Pathway

Nitric oxide is produced in endothelial cells by eNOS, which, in the presence of oxygen, NADPH and other cofactors, catalyses the oxidation of l-arginine to l-citrulline. NO diffuses into the underlying pulmonary vascular smooth muscle cells (PVSMC) and binds to soluble guanylate cyclase (sGC), which in turn, converts guanosine triphosphate (GTP) to cyclic guanosine monophosphate (cGMP). The subsequent activation of downstream cGMP-dependent protein kinases (PKG) results in pulmonary vasodilation. Additionally, NO inhibits PVSMC proliferation, platelet aggregation and thrombosis, collectively maintaining normal healthy pulmonary vasculature.

In PAH, there is decreased bioavailability of NO, causing vasoconstriction and increased smooth muscle cell proliferation, inflammation and thrombosis. Although these pathological changes were initially attributed to observed reductions of eNOS expression amongst PAH patients, more recent studies have demonstrated similar outcomes from persistent eNOS activation in mice and human models [27,29]. A potential explanation for this apparent contradiction is the role of reactive oxygen species (ROS), particularly tetrahydrobiopterin (BH_4_), in the enzymatic uncoupling of eNOS, thereby accounting for the pathogenesis of endothelial dysfunction, vasoconstriction and vascular remodelling in these models [30]. 

There are currently two approved drug classes acting on the nitric oxide pathway: phosphodiesterase 5 inhibitors (PDE-5i) and guanylate cyclase (GC) stimulators. PDE-5i prevent the degradation of cGMP, thereby increasing its plasma concentration and promoting the vasodilatory and antiproliferative effects of NO. GC stimulators act directly on sGC, even in the absence of NO, conferring similar increases in cGMP concentration.

### 4.2. Prostacyclin-Thromboxane A_2_ Pathway

Prostacyclins are produced in endothelial cells from arachidonic acid via cyclooxygenase and prostacyclin synthase. PGI_2_ binds to specific I-prostanoid (IP) receptors in smooth muscle cells, thereby activating adenylate cyclase. This enzyme converts adenosine triphosphate (ATP) to cyclic adenosine monophosphate (cAMP), which ultimately causes smooth muscle relaxation and subsequent vasodilation. Prostacyclin inhibits platelet aggregation, attenuates smooth muscle proliferation, and produces anti-inflammatory and antithrombotic effects. 

In PAH, the pathway shifts towards an alternative product, thromboxane A_2_, leading to platelet aggregation, vasoconstriction and proliferation [31]. The aetiological role of prostacyclin is evidenced by animal models, with IP-knockout mice exhibiting severe PAH and subsequent vascular remodeling when subjected to chronic hypoxia [32]. Furthermore, patients with PAH have reduced production of prostacyclins as well as reduced expression of prostacyclin receptor and prostacyclin synthase [33]. 

The clinical drugs developed for PAH therapy that act on the prostacyclin pathway are the prostacyclin analogues and receptor agonists. 

### 4.3. Endothelin-1 Pathway

Endothelin-1 (ET-1) is a peptide that acts as a potent vasoconstrictor [34]. ET-1 is produced on endothelial cell membranes from the precursor peptide big-endothelin-1 by endothelin-converting enzymes. ET-1 activates ET_A_ and ET_B_: two G-protein coupled receptors. ET_A_ is found on vascular smooth muscle cells, and promotes vasoconstriction, hypertrophy, proliferation, cell migration and fibrosis when activated. ET_B_ is located on both vascular smooth muscle and endothelial cell surfaces. On smooth muscle, activation of ET_B_ causes vasoconstriction whilst on endothelial surfaces, ET_B_ activates NO and prostacyclin production, causing vasodilation and anti-proliferation [35]. 

During PAH, there is an increase in expression of ET_A_ and smooth muscle ET_B_, but reduced expression of endothelial ET_B_ [35]. In addition, PAH patients exhibit increased ET-1 concentrations in their plasma and pulmonary vascular endothelial cells [36,37]. The pathological alterations to this pathway are counteracted by endothelin receptor antagonists (ERAs), which are available as ET_A_ selective or dual-action on ET_A_ and ET_B_ receptors.

## 5. Management of PAH

Treatment of PAH has progressed significantly over the past few decades in both its complexity and efficacy [38,39]. The aim of therapy is to achieve a low-risk status (maintaining WHO-FC II if possible) to preserve patient function, quality of life and minimise mortality risk [13]. This is generally achieved by optimising the patient’s six-minute walk distance (6MWD); however, current target thresholds rely upon cohort studies and expert consensus [40,41]. 

**Figure 2:**
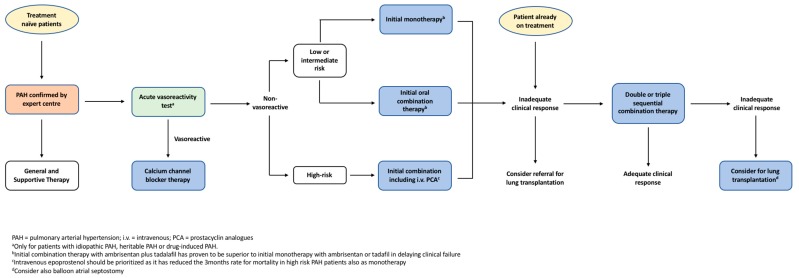
The European Society of Cardiology and the European Respiratory Society (ESC/ERS) evidence-based treatment algorithm for pulmonary arterial hypertension patients. Adapted from ECS/ERS guidelines for the diagnosis and treatment of pulmonary hypertension [13].

PAH management involves a stepwise pragmatic approach from general supportive treatment up to targeted pharmacological interventions as outlined in Figure 2. Selected patients undergo vasoreactivity testing, with those that exhibit sufficient vasodilation subsequently being commenced on regular high-dose calcium channel blockers (CCB) [13]. Patients who either fail to qualify for vasoreactivity testing or demonstrate inadequate response are then commenced on targeted monotherapy or combination therapies that counteract the pathophysiology of the condition.

### 5.1. General and Supportive Therapies

Supportive care remains the mainstay of PAH management despite insufficient and heterogeneous data supporting these practices [13]. These measures (supervised physical exercise, pregnancy avoidance, appropriate birth control, oxygen supplementation, diuretics, digoxin, oral anticoagulation, psychosocial support, genetic counselling) serve to provide symptomatic relief or minimise the morbidity associated with this condition.

Patients with PAH are recommended to engage in physical activity as permitted by their symptomatic control, with supervised rehabilitation performed at specialist centres for deconditioned patients [13]. The benefit of physical activity is elucidated by a randomised controlled trial (RCT) of 30 patients with optimal medical control, with the exercise group demonstrating improved exercise capacity, 6MWD, quality of life, functional status and oxygen consumption levels compared to non-training controls [42]. Subsequent RCTs have similarly found exercise training to improve fatigue levels, 6MWD and patient quality of life [43,44]. These recommendations are hampered by the limited study sample sizes (the largest having 183 patients), unknown prognostic implications and the lack of direct comparison between different exercise regimes.

Pregnancy, due to its associated high mortality and morbidity in PAH, is not recommended [13,45]. Additionally, pregnancy reduces the range of drugs available for PAH treatment since ERAs are formally contraindicated given their teratogenicity [46,47]. Recent registry data suggest treatment advancements may have partially ameliorated this burden, particularly amongst CCB-managed patients, but there are insufficient extensive, long-term trials to amend current pregnancy recommendations [48]. 

Appropriate birth control measures are an area of inquiry with less consensus. Barrier methods and progesterone are recommended and often used in combination, while oestrogens are used cautiously due to the risk of venous thromboembolism, pulmonary vascular effects and reduced efficacy due to drug–drug interactions with the ERA bosentan [49]. Should pregnancy continue despite education concerning high maternal and fetal risks, patients should receive disease-specific therapy and elective delivery with support from a PAH specialty team [50]. 

Hypoxia causes significant pulmonary arterial vasoconstriction and oxygen therapy has been demonstrated to reduce PVR in PAH patients [51]. However, there are no long-term randomised trials that evaluate the effects of oxygen, but expert consensus recommends sufficient supplementation to maintain saturation levels above 90% [52]. 

Optimising intravascular fluid status through the appropriate administration of diuretics reduces right ventricular (RV) dilatation, hepatic congestion, ascites and oedema. Despite this theoretical rationale, no RCTs have investigated the role of diuretics in PAH, thus the type and dosage of diuretics is largely left to clinical discretion [13]. 

Digoxin acutely improves cardiac output in iPAH patients, but its long-term use remains controversial since there is no data concerning chronic effects or clinical benefit [53]. Angiotensin converting enzyme-inhibitors, angiotensin receptor blockers, beta-blockers and ivabradine are not routinely recommended in the absence of associated comorbidities [13]. 

Given observed abnormalities in coagulation and fibrinolytic pathways in PAH patients, oral anticoagulation is currently recommended in patients with iPAH, hPAH or PAH secondary to anorexigens, based primarily upon short-term, single-centre retrospective studies [13,54,55,56,57,58]. However, the limited registry and RCT evidence available is inconclusive, heterogeneous and to-date has not examined the role of the new oral anticoagulant agents [54,57,58]. Anticoagulation is not recommended in other subtypes of PAH [13]. 

Multidisciplinary teams at specialised PH clinics provide a holistic approach to manage all aspects of patient care. Psychosocial counselling and patient support groups are effective for minimising the psychological, social, financial, spiritual and emotional impact on patients and their families [59,60]. As previously discussed, the option to provide genetic counselling to eligible patients is offered when medically and ethically appropriate [13]. 

### 5.2. Calcium Channel Blockers

Calcium channel blockers (CCB) inhibit the inflow of calcium into smooth muscle cells, thereby causing vasodilation. Most PAH patients demonstrate minimal response to acute vasodilatory challenges during right heart catheterisation testing; however, some patients demonstrate vasodilation to normal or near-normal pressures. In some of these patients, ongoing high-dose CCBs can have dramatic improvements with sustained haemodynamics to near physiological values [61]. Long-term response to CCB is most commonly observed in iPAH patients [61]. In contrast, patients with PAH associated with CTD, HIV, Portopulmonary hypertension (porto-pulmonary-HTN) and Pulmonary veno-occlusive disease (PVOD) exhibit poor long-term CCB efficacy, even in the presence of positive vasoreactivity testing [62]. Therefore, the ESC/ERS guidelines recommend pulmonary vasoreactivity testing in iPAH, hPAH and drug induced PAH patients [13]. Patients with insufficient responses to high-dose CCBs should be commenced on additional PAH-specific therapies [13]. CCBs should not be administered in patients with negative vasodilatory results or those who have not undergone vasoreactivity studies, due to potential side effects of RV failure, hypotension and syncope [13]. 

### 5.3. Targeted Therapies

Specific therapies which address the underlying pathophysiology of PAH include phosphodiesterase-5 inhibitors (PDE-5i), guanylate cyclase (GC) stimulators, prostacyclin analogues, prostacyclin receptor agonists, and endothelin receptor antagonists (ERA). In comparison to historical treatment regimes, these targeted therapies have revolutionised modern PAH management [38], with several studies having demonstrated improved exercise capacity, WHO-FC and time to clinical worsening (TTCW) as summarised in Table 3. The individual studies were not sufficiently powered to analyse mortality but a subsequent meta-analysis of RCTs demonstrated a 43% reduction in mortality rate [63].

### 5.4. Phosphodiesterase-5 Inhibitors

Sildenafil is a selective PDE-5i which is predominantly prescribed orally but can be administered intravenously for long-term patients who are unable to tolerate oral formulations. The SUPER-1 (Sildenafil Use in Pulmonary Arterial Hypertension) trial randomly allocated 278 treatment-naïve PAH patients to either placebo or sildenafil for 12 weeks [82]. Sildenafil conferred significant improvements in 6MWD, WHO-FC and pulmonary artery pressures when compared to placebo, but there was no difference in clinical worsening incidence [82]. When extended to three years in the SUPER-2 trial, 60% and 46% of patients maintained or improved their WHO-FC and 6MWD respectively [84]. Addition of sildenafil to epoprostenol similarly improved 6MWD and TTCW [76]. Some adverse effects of sildenafil included headaches, flushing, epistaxis, dyspepsia and diarrhoea, ranging from mild to moderate in severity [82]. Since PDE-5i cause vasodilation, these drugs should be used cautiously in combination with other vasodilatory agents, particularly nitrates and CCBs. 

Tadalafil, an alternative PDE-5i medication, possesses a superior pharmacokinetic profile in that it is dispensed once-daily in comparison to sildenafil which requires thrice-daily administration. In the PHIRST-1 (Pulmonary Arterial Hypertension and Response to Tadalafil) trial, 405 patients who were either treatment-naïve or on existing bosentan therapy, were randomised to receive placebo or tadalafil for 16 weeks [78]. The tadalafil arms of both the treatment-naïve and background therapy groups demonstrated significantly improved exercise capacity, symptomatic control, haemodynamics and reduced TTCW [78]. Tadalafil was well tolerated in the long-term, with sustained improvements in exercise capacity [85]. 

### 5.5. Guanylate Cyclase Stimulators 

Riociguat directly activates sGC, thus promoting vasodilation. In PATENT-1 (Pulmonary Arterial Hypertension Soluble Guanylate Cyclase-Stimulator Trial), 443 patients who were either treatment-naïve or had existing PAH treatment were randomised to placebo or riociguat, with the latter group demonstrating favourable outcomes regarding 6MWD, haemodynamics, WHO-FC and TTCW [77]. On sub-analysis, improved exercise capacity was also observed in patients on existing background therapy [77]. The long-term efficacy and safety profile of riociguat are currently being evaluated in PATENT-2, with interim analysis showing acceptable drug tolerability and continued improvements in 6MWD and WHO-FC compared to the PATENT-1 baseline [86]. The adverse effects of GC stimulators are similar to PDE-5i, including hypotension and syncope [77]. Combination therapy with PDE-5i was found to have a non-positive benefit-risk ratio due to increased hypotension and other drug-related side-effects [87]. 

### 5.6. Prostacyclin Analogues

Epoprostenol is a synthetic prostacyclin that can only be administered intravenously due to its short half-life (3–5 min) and limited stability at room temperature. Randomised trials have examined the benefit of continuous epoprostenol infusions in patients with iPAH [68,79] and PAH secondary to the scleroderma spectrum of disease [67], with this regimen producing improved symptomatic control, exercise capacity and haemodynamics [67,68,79]. Crucially, one study found improved survival rates among patients with severe iPAH—making epoprostenol the only therapy to-date to demonstrate a reduced mortality rate within a single RCT [68]. According to the ESC/ERS guidelines, a subsequent meta-analysis of the aforementioned randomised trials found a risk reduction in mortality of about 70% [13,67,68,79]. 

Treprostinil is a prostanoid analogue with similar properties to epoprostenol, but due to its longer half-life, it can be administered through multiple routes. A RCT comparing subcutaneous treprostinil to placebo demonstrated exercise capacity, symptomatic control and haemodynamics improved significantly, however infusion site pain was a common adverse event causing 8% of the population to withdraw from the study [81]. A RCT intended to investigate intravenous (IV) treprostinil was closed prematurely due to safety considerations [88]. Amongst the enrolled population, in 44 (35%) of the planned 126 participants, there were significant improvements in exercise capacity, symptoms and functional class [88]. Observational studies have found short-term IV treprostinil to be tolerable and produce a similar benefit profile to epoprostenol [89,90]. The TRIUMPH (Treprostinil Sodium Inhalation Used in the Management of Pulmonary Arterial Hypertension) trial investigated the addition of inhaled treprostinil to background therapy of either bosentan or sildenafil [83]. There was a significant improvement in 6MWD and quality of life, but minimal effect on functional class or TTCW [83]. The FREEDOM-C (Multicenter, Double-101 Blind, Randomized, Placebo-Controlled Study of the Efficacy and Safety of Oral Treprostinil Sustained Release Tablets in Subjects With Pulmonary Arterial Hypertension) [73] and FREEDOM-C2 [74] trials studied oral formulations of treprostinil in PAH patients on background bosentan and/or sildenafil therapy. Both studies demonstrated non-significant improvements in 6MWD [73,74] however a subsequent RCT with treatment-naïve patients observed a significant improvement [91]. 

Iloprost is available in IV, oral or aerosol formulations, although the effects of oral iloprost have not yet been studied. Inhaled iloprost, as demonstrated in the AIR (Aerosolized Iloprost Randomized) study, conferred significant improvements in symptomatic control, haemodynamics, WHO-FC and quality of life, though it was commonly associated with flushing and jaw pain [64]. Data concerning IV iloprost in PAH is scarce, with an observational study demonstrating limited clinical benefits [92]. 

### 5.7. Prostacyclin Receptor Agonists

Selexipag is an oral prostacyclin IP receptor agonist which produces vasodilatory and anti-proliferative effects. This agent was studied in the 71-week GRIPHON (Prostacyclin Receptor Agonist in Pulmonary Arterial Hypertension) study, which involved 1156 PAH patients with pre-existing monotherapy or dual therapy [75]. Time to first morbidity or mortality event was significantly reduced by 40%, though mortality rates were not significantly affected [75]. Selexipag significantly improved 6MWD, but was associated with headache, diarrhoea, nausea and jaw pain [75]. 

### 5.8. Endothelin Receptor Antagonists (ERA)

Bosentan is a dual-acting ERA, binding to both the ET_A_ and ET_B_ receptors. The BREATHE-1 (Bosentan Randomized Trial of Endothelin Antagonist Therapy) study commenced 213 treatment-naïve PAH patients on a 12-week bosentan regimen which conferred improvements in 6MWD, functional class and time to first worsening [69]. These findings were similarly demonstrated in a subsequent 24-week study, with both studies showing increased hepatic enzymes as the main adverse event [69,72]. COMPASS-2 is a more recent, 38-month trial involving 334 patients on background sildenafil therapy who were randomised to either bosentan or placebo [71]. This study did not meet its primary endpoint (time to first morbidity or mortality) nor its secondary endpoints (functional class and PAH hospital-related admissions) except for a significant improvement in 6MWD [71]. 

Ambrisentan selectively binds to ET_A_ receptors, with minimal binding to vasodilatory endothelial ET_B_ receptors [66]. ARIES-1 (Ambrisentan in Pulmonary Arterial Hypertension, Randomized, Double-Blind, Placebo-Controlled, Multicentre Efficacy Study) and ARIES-2 involved 202 and 192 treatment-naïve PAH patients respectively [66]. Ambrisentan elicited improvements in 6MWD, functional class and TTCW, but produced adverse effects including peripheral oedema, headache and nasal congestion [66]. 

Macitentan is a novel drug targeting ET_A_ and ET_B_ receptors. SERAPHIN (Study with an Endothelin Receptor Antagonist in Pulmonary Arterial Hypertension to Improve Clinical Outcome) compared macitentan to placebo in 742 PAH patients (who were either treatment-naïve or on background therapy) over an average of 100 weeks [80]. Macitentan significantly reduced the composite endpoint (morbidity and mortality events, PAH-related hospitalisations, 6MWD and WHO-FC), although the study was insufficiently powered to show significant mortality reduction in sub-analysis [80]. These benefits were observed in both treatment-naïve and background-treated patients [80]. 

### 5.9. Combination Therapies

Combination therapies have been increasingly recognised in the treatment of PAH as they allow the targeting of multiple signalling pathways. A meta-analysis performed by Galiè and colleagues showed a reduction in TTCW and improvements in clinical outcomes when sequential combination therapy was added following suboptimal response to monotherapy [13,93]. Although the meta-analysis demonstrated a reduction in mortality, the analysis was not sufficiently powered to achieve statistical significance [93]. A recent meta-analysis similarly demonstrated benefits of combination therapy over monotherapy [39]. 

BREATHE-2, an extension of BREATHE-1, was the first trial to explore combination therapy and involved 33 patients on existing epoprostenol treatment who were either randomised to placebo or bosentan [70]. BREATHE-2 failed to demonstrate significant differences in haemodynamics or clinical improvement, however these conclusions are limited by the study’s small sample size [70]. This has been investigated in subsequent studies, such as COMPASS-2 [71] and PACES [76], and some results can be inferred from subanalyses of other studies with patients on background therapy from baseline. Based on the findings of these studies, the ESC/ERS pulmonary hypertension guidelines outline several possible combination therapies for PAH [13]. 

The AMBITION (Ambrisentan and Tadalafil in Patients with Pulmonary Arterial Hypertension) trial was a pivotal study in combination therapy which directly investigated the role of initiating dual therapy in treatment-naïve patients by randomising these participants to either combination therapy or monotherapy of either ambrisentan or tadalafil for 73 weeks [65]. The primary endpoint (time to clinical failure event) was reached with both dual and monotherapy, with the former group exhibiting an additional 50% relative reduction compared to the latter [65]. This was the first study to provide a direct outcome comparison between monotherapy and combination therapy in treatment-naïve patients, thereby creating a new paradigm for the initial management of high-risk PAH.

### 5.10. Clinical Approach to PAH Treatment

The ESC/ERS guidelines recommend a stepwise pragmatic approach to PAH management as outlined in Figure 2 [13]. Recent long-term trials have revealed that targeted therapies confer significant improvements in clinical outcomes, thereby changing the therapeutic approach to PAH [39,63]. Risk assessment and treatment options should be considered at the time of diagnosis, with the aim of treatment to maintain low risk profiles [13]. Risk assessment, as discussed earlier, stratifies patients into low, intermediate or high risk of clinical worsening or death, although other prognostic factors should also be considered [13]. Patients with low or intermediate risk can be initiated on monotherapy, with regular monitoring of treatment response [13]. Results from the SERAPHIN and GRIPHON trials suggest there is benefit in adding either long-term macitentan or selexipag to background therapies in those patients who require additional treatment [75,80]. Conversely, for high-risk patients, the AMBITION trial highlighted the benefits of immediate combination therapy rather than a graduated, stepwise approach [65]. Often, IV epoprostenol is prioritised in high-risk patients given that this agent has been demonstrated to improve 3-month mortality rate of patients with severe PAH [13,68]. Patients refractory to treatment may be considered for lung transplantation or balloon atrial septostomy, the discussion of which exceeds the scope of this review.

Since bosentan induces cytochrome P450 isoenzymes CYP3A4 and CYP2C9, and sildenafil is metabolised by these same isoenzymes, care should you taken to avoid drug-drug interactions. Furthermore, PAH-targeted medications should be used cautiously in patients taking co-existing antihypertensives to avoid systemic hypotension.

### 5.11. Future Research

The past few decades have seen dramatic changes in the understanding and management of PAH, leading to improved symptomatic control, exercise tolerance and progression free survival [38,39,63]. However, there have been variable definitions in study endpoints—particularly TTCW, complicating its interpretation in meta-analyses [39]. This endpoint includes a collection of outcomes, including symptomatic worsening, lack of improvement, PAH-related hospital admissions, transplantation and mortality [94]. However, PAH-related hospitalisation, mortality and transplantation often follows clinical deterioration and hence TTCW may underestimate the true mortality and morbidity [39]. Further research should have a consistent definition in study endpoints and broader analysis of total PAH-related events [39,94]. Nevertheless, clinical worsening and mortality was still prevalent in these studies despite adequate therapy [39,63] indicating the need for identification of novel therapeutic agents. Research is currently underway to investigate the role of oestrogen [95], tyrosine kinase inhibitors [96] and BMPRII gene activation [97], (amongst many others) in the management of PAH. 

## 6. Conclusions

Substantial advancements in the understanding and management of PAH have been made in the recent decades. The current understanding of PAH aetiology includes the nitric oxide, prostacyclin-thromboxane and endothelin-1 pathways. This has led to five classes of drugs which target these the pathways: namely phosphodiesterase-5 inhibitors, soluble guanylate cyclase inhibitors, prostacyclin analogues, prostacyclin receptor agonists and endothelin receptor antagonists. Recent long-term trials have shown evidence of progression-free survival with initial, or early, addition of these drugs, either in isolation or in combination with other drug classes. In particular, emerging evidence has favoured the use of initial combination therapies in high risk patients. These medications are recommended with general and supportive measures; however, the evidence supporting these practices is heterogeneous and inconclusive. Ongoing study into alternative pathways involved in PAH pathogenesis represent opportunities for future targets in PAH management.

## Figures and Tables

**Table 1:** Classification of pulmonary hypertension [1].

**1** **Pulmonary arterial hypertension (PAH)** 1.1 Idiopathic PAH 1.2 Heritable PAH 1.2.1 BMPRII 1.2.2 ALK1, ENG, SMAD9, CAV1, KCNK3 1.2.3 Unknown 1.3 Drug and toxin induced 1.4 Associated with: 1.4.1 Connective tissue disease 1.4.2 HIV infection 1.4.3 Portal hypertension 1.4.4 Congenital heart diseases 1.4.5 Schistosomiasis 1′ Pulmonary veno-occlusive disease and/or pulmonary capillary haemangiomatosis 1′′ Persistent pulmonary hypertension of the newborn (PPHN)
**2** **Pulmonary hypertension due to left heart disease** 2.1 Left ventricular systolic dysfunction 2.2 Left ventricular diastolic dysfunction 2.3 Valvular disease 2.4 Congenital/acquired left heart inflow/outflow tract obstruction and congenital cardiomyopathies
**3** **Pulmonary hypertension due to lung diseases and/or hypoxia** 3.1 Chronic obstructive pulmonary disease 3.2 Interstitial lung disease 3.3 Other pulmonary diseases with mixed restrictive and obstructive pattern 3.4 Sleep-disordered breathing 3.5 Alveolar hypoventilation disorders 3.6 Chronic exposure to high altitude 3.7 Developmental lung diseases
**4** **Chronic thromboembolic pulmonary hypertension (CTEPH)**
**5** **Pulmonary hypertension with unclear multifactorial mechanisms** 5.1 Haematologic disorders: chronic haemolytic anaemia, myeloproliferative disorders, splenectomy 5.2 Systemic disorders: sarcoidosis, pulmonary histiocytosis 5.3 Metabolic disorders: glycogen storage disease, Gaucher disease, thyroid disorders 5.4 Others: tumoural obstruction, fibrosing mediastinitis, chronic renal failure, segmental PH

BMPRII = bone morphogenetic protein receptor type II; ALK1 = activin receptor-like kinase 1; ENG = endoglin 1; SMAD9 = mothers against decapentaplegic homolog 9; CAV1 = caveolin-1; KCNK3 = Potassium channel subfamily K member 3; HIV = human immunodeficiency virus.

**Table 2:** Prognostic risk stratification of pulmonary arterial hypertension. Adapted from 2015 ESC/ERS guidelines for the diagnosis and treatment of pulmonary hypertension [13].

Determinants of Prognosis	Low Risk: <5%	Intermediate Risk: 5–10%	High Risk: >10%
Clinical signs of RHF	Absent	Absent	Present
Progression of symptoms	No	Slow	Rapid
Episodes of syncope	None	Occasional ^a^	Recurrent ^b^
WHO functional class	I, II	III	IV
6-minute walk distance	>440 m	165–440 m	<165 m
Cardiopulmonary exercise testing	Peak VO_2_: >15 mL/min/kg (>65% pred.) VE/VCO_2_: slope < 36	Peak VO_2_: 11–15 mL/min/kg (35–65% pred.) VE/VCO_2_: slope 36–44.9	Peak VO_2_: <11 mL/min/kg (<35% pred.) VE/VCO_2_: slope ≥ 45
NT-proBNP plasma levels	BNP: <50 ng/L NT-proBNP: <300 ng/L	BNP: 50–300 ng/L NT-proBNP: 300–1400 ng/L	BNP: >300 ng/L NT-proBNP: >1400 ng/L
Imaging (Echocardiogram, CMR imaging)	RA area: <18 cm^2^ No pericardial effusion	RA area: 18–26 cm^2^ No or minimal pericardial effusion	RA area: >26 cm^2^ Pericardial effusion
Haemodynamics	RAP: <8 mmHg CI: ≥ 2.5 L/min/m^2^ SvO_2_: >65%	RAP: 8–14 mmHg CI: 2.0–2.4 L/min/m^2^ SvO_2_: 60–65%	RAP: >14 mmHg CI: <2.0 L/min/m^2^ SvO_2_: <60%

RHF = right heart failure; WHO = World Health Organisation; BNP = brain natriuretic peptide; NT-proBNP = N-terminal pro-brain natriuretic peptide; CMR = cardiac magnetic resonance; VO_2_ = oxygen consumption; pred = predicted; VE/VCO_2_ = minute ventilation/carbon dioxide production; RA = right atrium; RAP = right atrial pressure; CI = cardiac index; SvO_2_ = mixed venous oxygen saturation; ^a^ Occasional syncope—syncope that occurs only with high intensity exercise. ^b^ Recurrent syncope—syncope that occurs with moderate to low intensity exercise.

**Table 3:** Summary of randomised clinical trials of drugs approved for treatment of pulmonary arterial hypertension.

	Background Therapy	Number of Participants	Study Duration (Weeks)	Primary Endpoint	Secondary Endpoint	Main Adverse Events
AIR [64] (iloprost)	None	203	12	6MWD	NYHA functional class Mahler dyspnoea index Quality of life Death (NS)	Flushing Jaw pain
AMBITION [65] (ambrisentan vs. tadalafil vs. dual)	None	500	74	Time to first clinical failure	6MWD WHO-FC (NS) Borg dyspnoea index	Peripheral oedema Headache Nasal congestion
ARIES-1 [66] (ambrisentan)	None	202	12	6MWD	TTCW (NS) WHO-FC Quality of life (NS) Borg dyspnoea score BNP	Peripheral oedema Headache Flushing
ARIES-2 [66] (ambrisentan)	None	192	12	6MWD	TTCW WHO-FC (NS) Quality of life Borg dyspnoea score BNP	Peripheral oedema Headache Nasal congestion
Badesch and colleagues [67] (epoprostenol)	None	111	12	6MWD	Haemodynamics NYHA functional class	Jaw pain Diarrhoea Nausea and vomiting infection
Barst and colleagues [68] (epoprostenol)	none	81	12	6MWD	WHO-FCH aemodynamics (NS) Survival	Jaw pain Flushing Headaches Catheter related sepsis
BREATHE-1 [69] (bosentan)	None	213	12	6MWD	Borg dyspnoea index WHO-FC TTCW	Abnormal hepatic function
BREATHE-2 [70] (bosentan)	Epoprostenol	33	16	TPR (NS)	CI (NS) PVR (NS) 6MWD (NS) WHO-FC (NS)	Mainly related to epoprostenol therapy
COMPASS-2 [71] (bosentan)	Sildenafil	334	38 months	Time to first morbidity or mortality event (NS)	6MWD WHO-FC (NS) PAH-related admissions (NS)	Abnormal hepatic function
EARLY [72] (bosentan)	None or Sildenafil	185	24	6MWD (NS) PVR	TTCW WHO-FC Quality of life	Abnormal liver function test
FREEDOM-C [73] (treprostinil)	ERA and/or PDE-5i	350	16	6MWD (NS)	Clinical worsening (NS) WHO-FC (NS) Borg dyspnoea score (NS)	Headache Nausea and vomiting Diarrhoea Flushing Jaw pain
FREEDOM-C2 [74] (treprostinil)	ERA and/or PDE-5i	310	16	6MWD (NS)	Clinical worsening (NS) WHO-FC (NS)	Headache Nausea and vomiting Diarrhoea Flushing Jaw pain
GRIPHON [75] (selexipag)	ERA and/or PDE-5i	1156	71	Event point event	6MWD WHO-FC	Headache Jaw pain Flushing Diarrhoea
PACES [76] (sildenafil)	Epoprostenol	267	16	6MWD	TTCW WHO-FC	Headache Dyspepsia
PATENT-1 [77] (riociguat)	None or ERA or PCA	443	12	6MWD	PVR Borg dyspnoea score WHO-FC	Headache Dyspepsia Hypotension
PHIRST [78] (Tadalafil)	None or Bosentan	405	16	6MWD	WHO-FC (NS) TTCW Quality of life	Headache Myalgia Flushing
Rubin and colleagues [79] (epoprostenol)	None	23	12	6MWD	Haemodynamics	Diarrhoea Jaw pain Photosensitivity
SERAPHIN [80] (macitentan)	None or PDE-5i or PCA	742	100	Time to first event	6MWD WHO-FC PAH related admissions	Nasopharyngitis Headache Anaemia
Simonneau and colleagues [81] (treprostinil)	None	470	12	6MWD	Symptoms Borg dyspnoea score Haemodynamics	Infusion site pain Jaw pain Diarrhoea
SUPER [82] (sildenafil)	None	278	12	6MWD	WHO-FC TTCW (NS) Haemodynamics	Flushing Dyspepsia Diarrhoea
TRIUMPH [83] (treprostinil)	ERA or PDE-5i	235	12	6MWD	Quality of life TTCW (NS) Symptoms (NS)	Cough Headache Flushing

NS = not statistically significant; 6MWD = placebo corrected 6-minute walk distance; TTCW = time to clinical worsening; WHO-FC = World Health Organisation Functional Class; NYHA = New York Heart Association BNP = brain natriuretic peptide; PVR = pulmonary vascular resistance; TPR = total pulmonary resistance; CI = cardiac index; ERA = endothelin receptor antagonists; PDE-5i = phosphodiesterase type-5 inhibitors; PCA = prostacyclin receptor agonist; PAH = pulmonary arterial hypertension.
